# Long Term Clinical Prognostic Factors in Relapsing-Remitting Multiple Sclerosis: Insights from a 10-Year Observational Study

**DOI:** 10.1371/journal.pone.0158978

**Published:** 2016-07-08

**Authors:** Gabriel Bsteh, Rainer Ehling, Andreas Lutterotti, Harald Hegen, Franziska Di Pauli, Michael Auer, Florian Deisenhammer, Markus Reindl, Thomas Berger

**Affiliations:** 1 Clinical Department of Neurology, Medical University of Innsbruck, Innsbruck, Austria; 2 Department of Neurology, University Hospital Zurich & University of Zurich, Zurich, Switzerland; University of Muenster, GERMANY

## Abstract

**Background:**

Multiple sclerosis (MS) has a highly heterogenic course making prediction of long term outcome very difficult.

**Objective:**

The objective was to evaluate current and identify additional clinical factors that are linked to long term outcome of relapsing-remitting MS assessed by disability status 10 years after disease onset.

**Methods:**

This observational study included 793 patients with relapsing-remitting MS. Clinical factors hypothesized to influence long term outcome measured by EDSS scores 10 years after disease onset were analysed by Kaplan-Meier-estimates. Multinomial logistic regression models regarding mild (EDSS ≤2.5), moderate (EDSS 3.0–5.5) or severe (EDSS ≥6.0) disability were calculated to correct for confounders.

**Results:**

Secondary progression was the strongest predictor of severe disability (Hazard ratio [HR] 503.8, 95% confidence interval [CI] 160.0–1580.1); p<0.001). Complete remission of neurological symptoms at onset reduced the risk of moderate disability (HR 0.42; CI 0.23–0.77; p = 0.005), while depression (HR 3.59; CI 1.14–11.24; p = 0.028) and cognitive dysfunction (HR 4.64; CI 1.11–19.50; p = 0.036) 10 years after disease onset were associated with severe disability. Oligoclonal bands and pregnancy were not correlated with disability.

**Conclusion:**

We were able to identify clinically apparent chronic depression and cognitive dysfunction to be associated with adverse long term outcome in MS and to confirm that pregnancy has no negative impact. Additionally, we emphasize the positive predictive value of complete remission of initial symptoms.

## Introduction

Multiple Sclerosis (MS) is the most common cause of irreversible neurological disability in young adults with a prevalence of 120 per 100.000 [1]. MS is recognized as a chronic autoimmune disease of the central nervous system (CNS) causing demyelination and axonal loss [2].

The disease course of MS is either relapsing-remitting (RRMS) or chronic progressive (primary, PPMS, or secondary progressive, SPMS). Secondary progressive disease course poses the major risk for the majority of RRMS patients to accumulative permanent disability after a variable period of time [3].

Despite the therapeutic merit of a meanwhile broad armamentarium of disease modifying therapies (DMT) in reducing relapse rates in RRMS, it is still controversially discussed whether these drugs are really able to modify the long term disease course of MS, i.e. preventing or delaying conversion to SPMS.

In addition, prediction of long term outcome in patients with relapsing-remitting MS is of utmost importance ever since to enable individual prognosis and, thus, treatment decisions/adjustments. Over the past decades some clinical and magnetic resonance imaging (MRI) factors seem to be predictive for long term outcomes: male gender, higher age and pyramidal symptoms at disease onset, incomplete recovery from first clinical attack, higher baseline T2 lesion load, early brain atrophy, shorter time-interval to second clinical attack, number of relapses in early disease phase and–most importantly–development of progressive disease course. However, the heterogeneity of individual disease courses makes prognostic statements at disease onset highly difficult [3–6].

The objective of this 10 years observational study was to evaluate existing clinical prognostic factors and to possibly identify additional clinical risk factors associated with long term outcome in a large cohort of RRMS patients.

## Patients and Methods

### Data collection and patient population

In 2004 an electronic database was established at the MS Clinic of the Department of Neurology, Medical University of Innsbruck, which serves as both a primary and a reference centre mainly for western Austria and its geographical catchment area. The MS population is mainly of Caucasian ethnicity. From January 2^nd^ 2000 to July 13^th^ 2013, a cohort of 1601 MS patients according to Poser’s or McDonalds diagnostic criteria has been included in this database [7–9]. The prevalence of MS in Austria is 98 per 100.000 people [10]. Given a population of about 1.6 million people in western Austria, this study is likely to have caught most of MS patients from this geographic area [11].

Data were collected retrospectively at first visit and prospectively whenever the patient returned for scheduled (usually every 3–6 months, at least once yearly) follow-up or unscheduled visits. Database case reports include demographic data, smoking habits, family history, medical history, pregnancies, details of MS course (timepoint of first symptoms, time to diagnosis, relapses, number of relapses per year, Expanded Disability Status Scale [EDSS], standard relapse treatment, relapse outcome, onset of SPMS), occurrence of depressive symptoms or cognitive dysfunction, diagnostic data (MRI, cerebrospinal fluid findings, evoked potentials) and previous DMT, including initiation, interruption, change, and adverse effects of DMT.

Confidentiality and data protection are ensured in keeping with the recommendations of the declaration of Helsinki and the Austrian Data Safety Authority instructions. The study was approved by the ethics committee of the Medical University Innsbruck. Written informed consent was obtained from every patient at first visit.

### Definition of cases and assessment of patients

**Fig 1:**
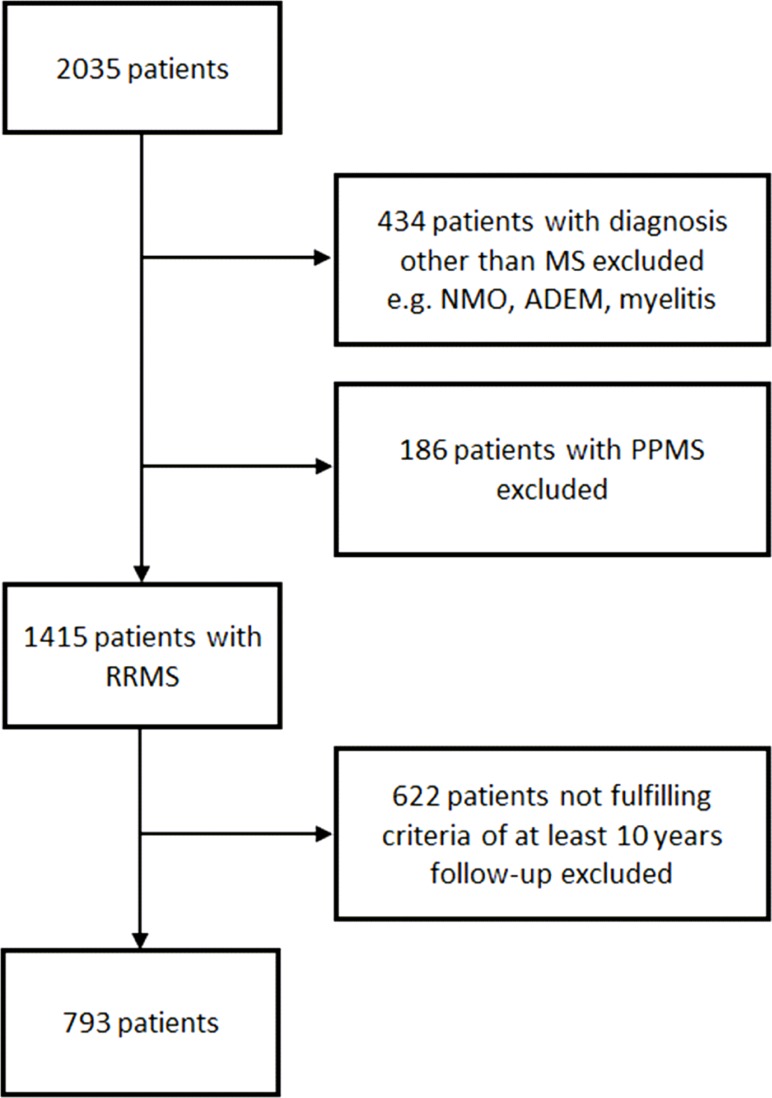
Inclusion flow chart. Abbreviations: ADEM = acute demyelinating encephalomyelitis; MS = multiple sclerosis; NMO = neuromyelitis optica; PPMS = primary progressive MS; RRMS = relapsing-remitting MS

For the present study, we included those 793 patients with onset of relapsing-remitting MS (RRMS) and at least 10 years of complete documented follow-up from onset of disease. (Fig 1)

The disease course was defined according to the classification of Lublin and Reingold [12]. A relapse was defined as patient-reported symptoms or objectively observed signs typical of an acute CNS inflammatory demyelinating event, current or prior to the visit, with duration of at least 24 hours in the absence of increased body temperature or infection, separated from the last relapse by at least 30 days [7]. SPMS was defined as sustained worsening of neurological symptoms (accounting for an EDSS progression of at least 1.0 point) for a duration of at least 12 months not related to a relapse and had to be confirmed in our MS Clinic [13]. EDSS score was recorded at each visit. To avoid documentation bias due to EDSS changes along with relapses, EDSS scores were only considered if they were confirmed after 6 months. MS onset symptoms were categorized according to Kurtzke’s Functional Systems (FS) [14]. Relapse treatment and degree of remission of onset symptoms after 6 months were also obtained. A history of depression and cognitive dysfunction before onset or its worsening or new occurrence after diagnosis of MS was rigorously asked and documented at each visit by the treating physician. Since not all patients included in the study had received formal neuropsychological testing for depression and cognitive dysfunction and because the testing methods used changed significantly over the period of observation making comparisons impossible, we decided to use clinical definitions of depression and cognitive dysfunction rather than formal testing. Depression was defined as a state of low mood) and loss of activity along with characteristic symptoms such as sadness, anxiety, awkwardness, loss of appetite, insomnia, up to suicidal thoughts. When cognitive dysfunction was reported by a patient or clinically suspected (by characteristic symptoms such as impaired memory, decreased attention or concentration, difficulties in orientation, learning, calculating, planning or performing any other cognitive task). To validate these definitions, we conducted correlation analyses comparing the accordance rate of clinical and formal diagnosis in the subgroup of patients who received formal neuropsychological testing (S1 Table). Depression testing was available for 140/793 patients and cognitive testing for 86/793 patients. We found strong accordance rates for the clinical diagnoses of depression (kappa 0.923; p<0.001) and cognitive dysfunction (kappa 0.884; p<0.001) compared to formal neuropsychological testing.

### Statistical Analysis

Statistical analysis was performed using SPSS 22.0 (SPSS Inc, Chicago, IL). Categorical variables were expressed in frequencies and percentages, and parametric continuous variables as mean and standard deviation (SD) or nonparametric variables as median and range.

Primary endpoint was EDSS score assessed 10 years after disease onset. We defined three groups designated to “mild” (EDSS 0–2.5), “moderate” (EDSS 3.0–5.5) or “severe” (EDSS 6.0 or higher) disability. Annualized relapse rates (ARR) were calculated for four time intervals 1) first year after onset (ARR y1), 2) second year after onset (ARR y2), 3) third to fifth year after onset (ARR y3-5) and 4) sixth to tenth year after onset (ARR y6-10) by dividing the number of relapses occurred by the respective time intervals. Comparisons were made by analysis of variance (ANOVA) for independent samples and Chi-Square tests as appropriate. A two tailed p-value < 0.05 was considered significant. Survival analyses were performed using Kaplan-Meier-estimates and log-rank-tests for comparison of prognostic factors over time. Multinomial logistic regression models were calculated regarding the primary endpoint (mild vs. moderate vs. severe disability) to evaluate the prognostic impact of the factors included (age at manifestation of patients’ first symptoms, sex, initial symptoms, remission of initial symptoms, presence of oligoclonal bands (OCB) in the CSF at time of diagnosis, ARR y1, ARR y2, ARR y3-5, ARR y6-10, development of SPMS, occurrence of depression, cognitive dysfunction, and pregnancy during the ten-year observation period).

## Results

### Basic characteristics of patients

793 relapsing-remitting MS (RRMS) patients fulfilled the inclusion criteria and were included in the analysis (Fig 1). Demographic and baseline clinical data as well as correlation with EDSS outcome 10 years after disease onset are shown in Table 1. The mild disability group after 10 years disease course had a significantly younger age at onset compared to the moderate and severe group (27.36 vs. 29.81/32.44; p = 0.006/p<0.001). There were no significant group differences regarding sex, family history of MS, presence of OCB in CSF or smoking before and after onset, although there were trends towards more male patients in the severe group and impact of smoking on further disease progression.

**Table 1:** Baseline clinical and demographic data and their correlation with EDSS outcome 10 years after disease onset

	Overall n = 793	Mild disability(EDSS 0–2.5) n = 583	Moderate disability(EDSS 3–5.5) n = 132	Severe disability (EDSS 6–10) n = 78	P value mild vs. moderate disability	P value mild vs. severe disability	P value moderate vs. severe disability	P value Overall
**Type of MS**^**a**^								
RRMS	650 (82.0)	568 (97.4)	76 (57.6)	6 (7.7)	6.0*10^−42^	1.1*10^−105^	2.2*10^−11^	1.1*10^−93^
SPMS	143 (18.0)	15 (2.8)	56 (42.4)	72 (92.3)				
**Time to diagnosis (years)**^**b**^	2.43 ± 3.29	2.50 ± 3.47	2.64 ± 3.03	1.57 ± 2.02	0.999	0.058	0.069	0.047
**Female**^**a**^	577 (72.8)	426 (73.1)	97 (73.5)	54 (69.2)	0.509	0.278	0.306	0.758
**Age at onset (years)**^**b**^	28.27 ± 8.41	27.36 ± 8.01	29.81 ± 7.98	32.44 ± 10.29	0.006	3.4*10^−5^	0.079	1.9*10^−7^
**Family History of MS**^**a**^	115 (14.5)	79 (13.6)	27 (20.5)	9 (11.5)	0.103	0.999	0.259	0.105
**Smoking before onset**^**a**^	240 (30.3)	167 (28.6)	46 (34.8)	27 (34.6)	0.999	0.999	0.999	0.215
**Smoking after onset**^**a**^	211 (26.6)	150 (25.7)	39 (29.5)	22 (28.2)	0.999	0.999	0.999	0.632
**Onset symptoms**^**a**^								
Visual	209 (26.4)	154 (26.4)	38 (28.8)	17 (21.8)	0.999	0.999	0.999	0.999
Brainstem	181 (22.8)	131 (22.5)	30 (22.7)	20 (25.6)	0.999	0.999	0.999	0.999
Pyramidal	177 (22.3)	106 (18.2)	37 (28.0)	34 (43.6)	0.071	3.0*10^−6^	0.048	1.9*10^−5^
Cerebellar	70 (8.8)	41 (7.0)	18 (13.6)	11 (14.1)	0.104	0.254	0.999	0.096
Sensory	381 (48.0)	288 (49.4)	65 (49.2)	28 (35.9)	0.999	0.132	0.328	0.616
Bladder/Bowel	19 (2.4)	8 (1.4)	7 (5.3)	4 (5.1)	0.088	0.340	0.999	0.056
Cerebral	6 (0.8)	2 (0.3)	4 (3.0)	0 (0.0)	0.999	0.999	0.999	0.999
Other	2 (0.3)	2 (0.3)	0 (0.0)	0 (0.0)	0.999	0.999	0.999	0.999
Polysymptomatic	208 (26.2)	131 (22.5)	46 (34.8)	31 (39.7)	0.008	0.003	0.858	7.1*10^−4^
**Steroid treatment received at manifestation of first symptoms**^a^*	349 (47.4)	267 (49.0)	51 (42.5)	31 (43.1)	0.352	0.617	0.999	0.972
**Oligoclonal bands at time of diagnosis**	753 (95.0)	552 (94.7)	126 (95.5)	75 (96.2)	0.825	0.220	0.602	0.533
**Complete Remission of onset symptoms**^**a**^	590 (77.6)	476 (84.4)	75 (61.0)	39 (53.4)	8.1*10^−8^	2.8*10^−8^	0.563	4.3*10^−13^

a…absolute numbers and percentages

b…mean ± standard deviation

p-values corrected for multiple testing (Bonferroni)

* standard treatment with 1000mg methylprednisolone for 3–5 days

Abbreviations: EDSS = Expanded Disability Status Scale; RRMS = relapsing remitting MS; SPMS = secondary progressive MS

A total of 593 (74.8%) patients had received one or more DMT during the observational period. Treated patients tended to have a higher relapse frequency and a more severe initial course of disease compared to the untreated group of patients. However, treatments were heterogeneous, mean duration of treatment was just 2.25 years (SD 2.84) and mean time to initiation of DMT was 6.63 years (SD 3.66). (S2 Table)

### Initial symptoms, remission, and oligoclonal bands

According to Kurtzke’s FS, 26.4% of patients had visual symptoms, 22.8% brainstem, 22.3% symptoms of the pyramidal tract, 8.8% cerebellar, 48.0% sensory, and 2.4% bladder/bowel symptoms at onset (Table 1). 26.2% of our patients had initial symptoms affecting more than one FS. Patients with a pyramidal or polysymptomatic onset symptom(s) were significantly less likely to have mild disability after ten years MS disease course. Complete remission of onset symptoms was achieved in 84.4% of the mild EDSS group compared to only 61.0% (p<0.001) in the moderate and 53.4% (p<0.001) in the severe disability group. There was no significant association between use of steroid treatment and the proportion of patients achieving complete remission of onset symptoms (p = 0.232). Overall, 95% of patients exhibited OCB positivity with no significant differences between the disability groups. (Table 1)

### Relapses and secondary progression

Mean time to second clinical attack was significantly longer in the mild compared to the moderate and severe disability group (4.1 vs. 2.7 and 2.0 years; p<0.001). Similar to the onset symptom analyses, the mild disability group had significantly less pyramidal (20.1%) and polysymptomatic (23.9%) symptoms at second clinical attack than the moderate (38.4%/39.2%; p<0.001) and severe (62.7%/45.3%; p<0.001) disability group. Likewise, complete remission of the second clinical attack showed a significant association with only mild disability after ten years. Annualized relapse rates (ARR) were significantly lower in the mild compared to the moderate outcome group except for the second year after onset. Comparing the mild and severe disability group, we found ARR to be associated with the endpoint in the first five years from onset but not afterwards. The cumulative number of relapses in the 10 years observation period was significantly different between the mild disability (3.74 relapses), moderate (5.24; p<0.001) and severe disability (4.99; p = 0.002) groups. 18% of all patients converted to SPMS during the 10 years of follow-up. However, only 2.6% in the mild, but 42.4% of the moderate and 92.3% of the severe disability group developed SPMS (p<0.001). Time to SPMS was markedly shorter in the severe compared to the mild and moderate outcome groups (4.9 vs. 10.0 and 8.6 years; p<0.001) Age at SPMS conversion tended to be lower in the severe and moderate EDSS groups as opposed to the mild EDSS group. (Table 2)

**Table 2:** Variables of disease course and their correlation with EDSS outcome 10 years after disease onset

	Overall n = 793	Mild disability(EDSS 0–2.5) n = 583	Moderate disability(EDSS 3–5.5) n = 132	Severe disability (EDSS 6–10) n = 78	P value mild vs. moderate disability	P value mild vs. severe disability	P value moderate vs. severe disability	P value Overall
**Second clinical attack**^**a**^	693 (87.4)	493 (84.6)	125 (94.7)	75 (96.2)	0.001	0.002	0.454	2.9*10^−8^
**Time to second clinical attack (years)**^**b**^	3.63 ± 3.14	4.06 ± 3.33	2.70 ± 2.31	2.01 ± 1.72	2.8*10^−5^	5.6*10^−6^	0.368	6.0*10^−10^
**Symptoms at second clinical attack**								
Pyramidal^a^	194 (28.0)	99 (20.1)	48 (38.4)	47 (62.7)	8.4*10^−5^	7.9*10^−13^	0.002	9.6*10^−15^
Polysymptomatic^a^	201 (29.0)	118 (23.9)	49 (39.2)	34 (45.3)	0.002	4.6*10^−4^	0.721	4.5*10^−5^
Bladder/bowel^a^	29 (4.2)	11 (2.2)	10 (8.0)	8 (10.7)	0.004	0.004	0.999	5.9*10^−4^
**Steroid treatment received at second clinical attack**^**a**^ *	458 (71.3)	326 (71.3)	81 (69.8)	51 (73.9)	0.999	0.999	0.999	0.999
**Complete Remission after second clinical attack**^**a**^	484 (74.1)	391 (84.1)	66 (56.9)	27 (37.5)	1.8*10^−8^	2.5*10^−14^	0.026	6.4*10^−19^
**Annualized relapse rate (ARR)**								
ARR y1^b^	0.29 ± 0.65	0.24 ± 0.55	0.45 ± 0.94	0.43 ± 0.70	0.002	0.040	0.999	3.7*10^−4^
ARR y2^b^	0.36 ± 0.65	0.31 ± 0.62	0.42 ± 0.66	0.63 ± 0.82	0.339	2.9*10^−4^	0.079	2.9*10^−4^
ARR y3-5^b^	0.36 ± 0.45	0.31 ± 0.42	0.49 ± 0.55	0.50 ± 0.47	2.1*10^−4^	0.002	0.999	7.0*10^−6^
ARR y6-10^b^	0.28 ± 0.32	0.26 ± 0.31	0.38 ± 0.37	0.29 ± 0.32	2.3*10^−4^	0.999	0.165	3.8*10^−4^
**Cumulated number of relapses**^**b**^	4.10 ± 2.93	3.74 ± 2.67	5.24 ± 3.64	4.99 ± 2.88	8.3*10^−7^	0.002	0.999	5.3*10^−8^
**Secondary Progression**								
**SPMS**^**a**^	143 (18.0)	15 (2.6)	56 (42.4)	72 (92.3)	1.2*10^−32^	5.2*10^−73^	1.2*10^−13^	6.0*10^−96^
**Time to SPMS (years)**^**b**^	9.24 ± 1.97	9.95 ± 0.43	8.62 ± 2.22	4.93 ± 2.69	8.4*10^−25^	1.5*10^−144^	1.4*10^−71^	4.1*10^−96^
**Age at SPMS (years)**^**b**^	38.00 ± 9.56	42.52 ± 8.58	38.23 ± 8.23	36.87 ± 10.52	0.364	0.113	0.999	0.111
**Depression**								
**Depression before onset**^**a**^	9 (1.1)	4 (0.7)	4 (3.0)	1 (1.3)	0.103	0.189	0.147	0.153
**Depression after onset**^**a**^	87 (11.0)	53 (9.1)	13 (9.8)	21 (26.9)	0.999	3.6*10^−5^	0.005	1.5*10^−5^
**Time to depression (years)**^**b**^	9.21 ± 2.35	9.34 ± 2.21	9.31 ± 2.16	8.07 ± 3.24	0.999	6.7*10^−5^	0.002	1.1*10^−4^
**Cognitive dysfunction** ^**a**^	42 (5.3)	19 (3.3)	8 (6.1)	15 (19.2)	0.293	7.3*10^−4^	0.142	9.2*10^−5^
**Time to cognitive dysfunction (years)**^**b**^	9.72 ± 1.41	9.79 ± 1.26	9.67 ± 1.51	9.25 ± 2.07	0.999	0.005	0.116	0.006
**Pregnancy**^**a**^	331 (57.4)	248 (58.2)	56 (57.7)	27 (50.0)	0.999	0.999	0.999	0.810
**Number of pregnancies**^**b**^	1.90 ± 0.98	1.84 ± 0.91	2.02 ± 1.00	2.19 ± 1.44	0.636	0.237	0.999	0.128

a…absolute numbers and percentages

b…mean ± standard deviation

p-values corrected for multiple testing (Bonferroni)

* standard treatment with 1000mg methylprednisolone for 3–5 days

Abbreviations: EDSS = Expanded Disability Status Scale; MS = multiple sclerosis; RRMS = relapsing-remitting MS; SPMS = secondary progressive MS

### Depression, cognitive dysfunction, and pregnancy

**Fig 2:**
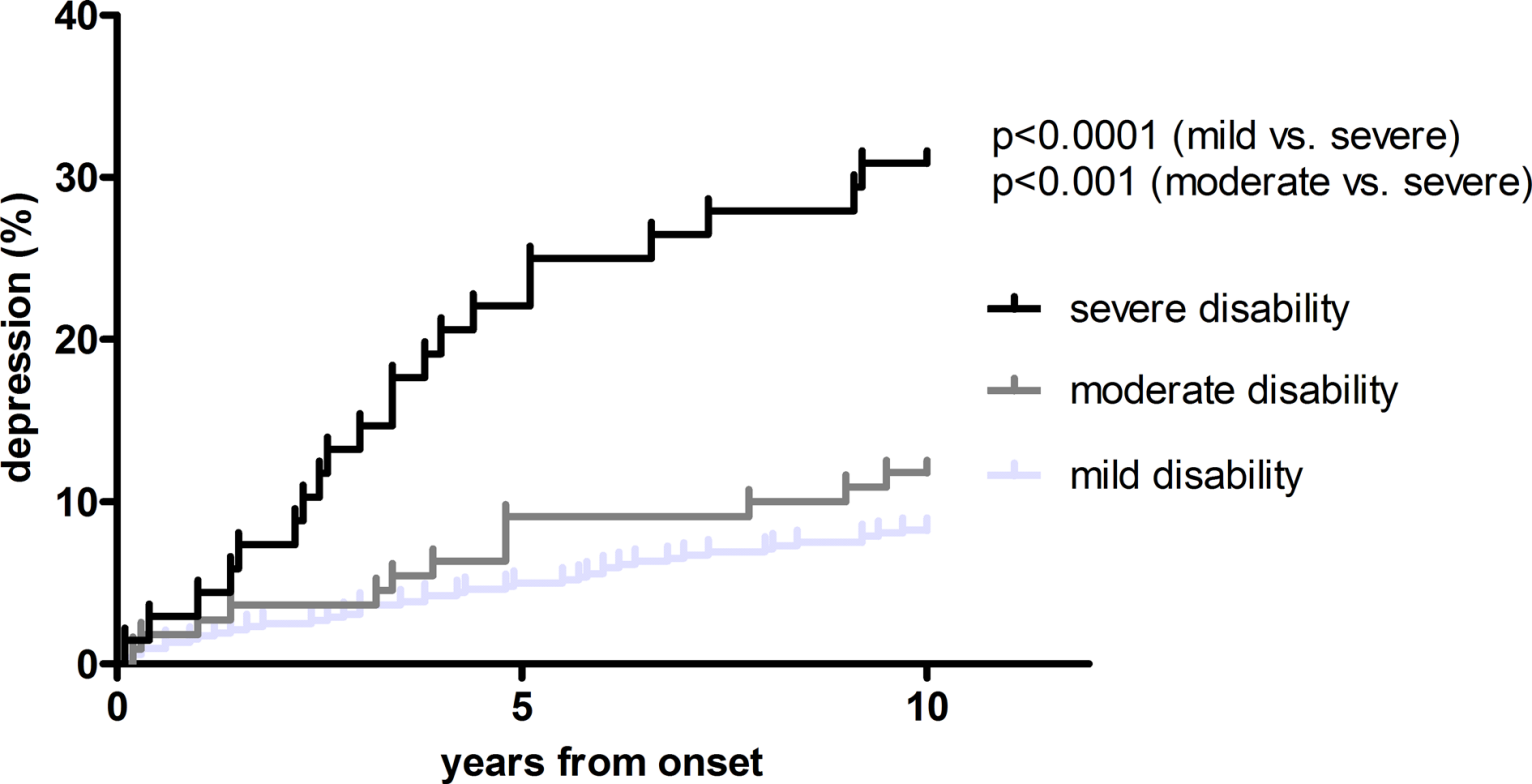
time to depression. Kaplan-Meier curves of the risk of developing depression according to disability status 10 years after onset. Log rank test used for calculation of significancy.

Eleven percent of RRMS patients were diagnosed with depression after MS onset and 4.8% developed cognitive dysfunction during the 10 years observation period. New onset depression occurred in 26.9% in the severe compared to 9.1% in the mild (p<0.001) and 9.8% in the moderate disability group (p = 0.005). Time to depression took 8.07 years in the severe, while it was 9.34 years in the mild (p<0.001) and 9.31 years in the moderate outcome group (p = 0.002). (Table 2, Fig 2).

**Fig 3:**
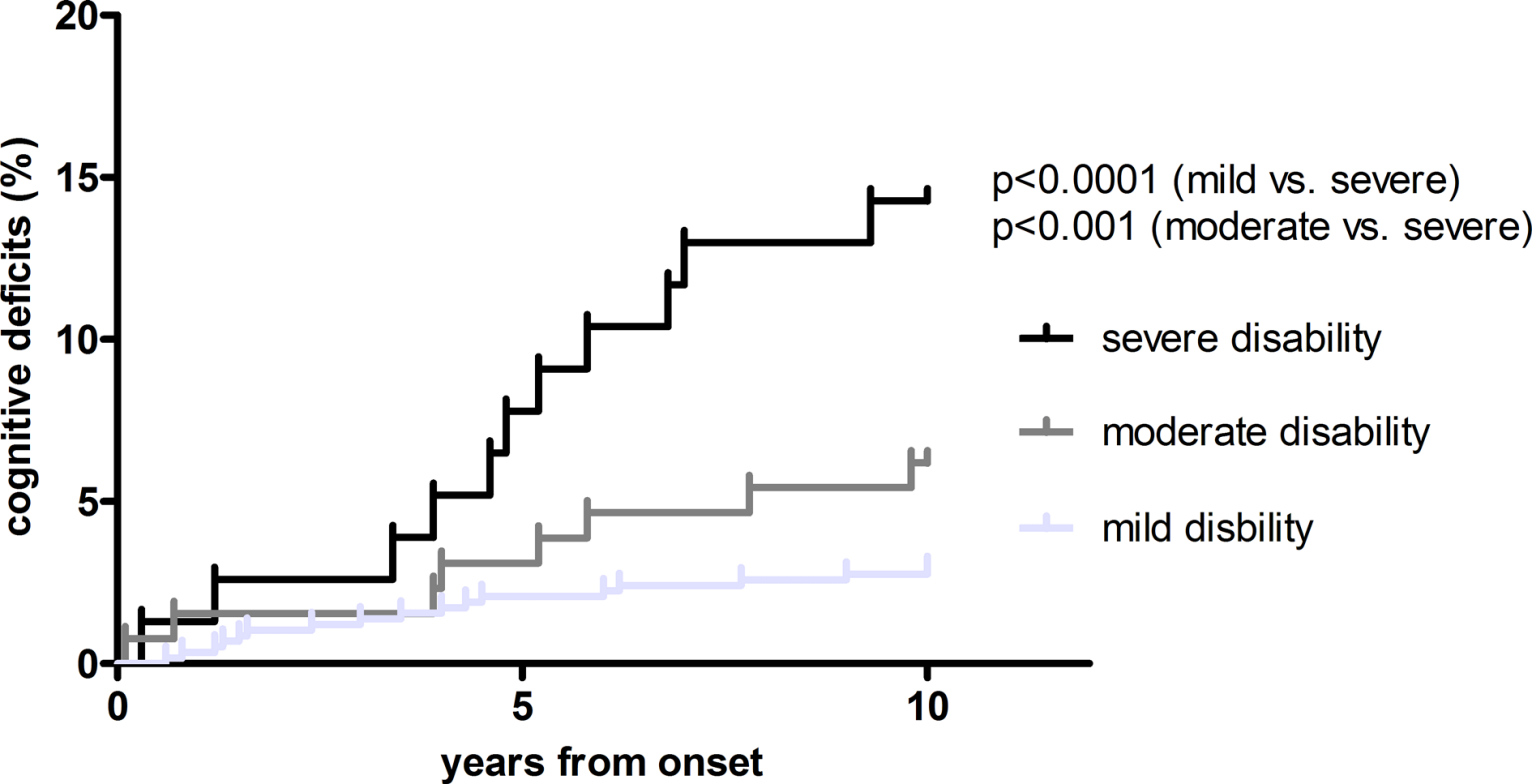
time to cognitive dysfunction. Kaplan-Meier curves of the risk of developing cognitive dysfunction according to disability status 10 years after onset. Log rank test used for calculation of significancy.

Cognitive dysfunction was diagnosed in 14.1% of severely disabled patients, but was present in only 3.3% and 6.1% of mildly and moderately disabled patients, respectively. Correspondingly, time to cognitive dysfunction was notably shorter in the severe (9.25 years) than in mild disability group (9.79 years; p = 0.005). (Fig 3)

Out of 577 female patients, 331 (57.4%) became pregnant at least once during the first ten years after disease onset. There were no significant differences between the three disability outcome groups regarding any pregnancy related data. (Table 2)

### Multivariate analysis

Multinomial logistic regression was performed to identify clinical factors for prognosis of disability 10 years after disease onset (Table 3). Overall, the model revealed occurrence of secondary progressive disease course, pyramidal symptoms at disease onset, number of relapses, development of depression and incomplete remission of onset symptoms as significant risk factor for poor outcome.

**Table 3:** Prognostic factors in RRMS according to EDSS outcome 10 years after disease onset.

	mild vs. moderate disability		mild vs. severe disability		overall
	Hazard ratio (CI)	P value	Hazard ratio (CI)	P value	P value^b^
Female	1.13 (0.62–2.05)	0.698	1.30 (0.52–3.28)	0.578	0.845
Age at manifestation of patients’ first symptoms	1.03 (0.99–1.06)	0.130	1.03 (0.99–1.08)	0.176	0.252
Pyramidal initial symptoms	1.36 (0.74–2.49)	0.324	3.44 (1.35–8.76)	0.010	0.033
Complete remission	0.42 (0.23–0.77)	0.005	0.65 (0.25–1.65)	0.361	0.021
Oligoclonal bands	1.05 (0.99–1.08)	0.647	1.09 (0.97–1.11)	0.428	0.769
Number of relapses	1.13 (1.07–1.19)	7.0*10^−6^	1.07 (0.97–1.18)	0.160	5.1*10^−5^
Secondary progression	29.99 (13.96–64.41)	2.8*10^−18^	503.8 (160.0–1580.1)	1.8*10^−26^	9.6*10^−58^
Depression	0.76 (0.33–1.76)	0.525	3.59 (1.14–11.24)	0.028	0.012
Cognitive dysfunction	2.48 (0.84–7.30)	0.099	4.64 (1.11–19.50)	0.036	0.102
Pregnancy^a^	0.87 (0.48–1.58)	0.647	0.68 (0.26–1.78)	0.434	0.725

Multinominal logistic regression. Reference category is mild disability 10 years after disease onset. A hazard ratio (HR) > 1 indicates that the variable is associated with a higher risk of having moderate or severe disability compared to the reference group (mild disability).

Numbers in brackets show 95% confidence interval (CI) for HR.

a…this analysis was only conducted for female patients

b…likelihood ratio tests

Abbreviations: EDSS = Expanded Disability Status Scale; RRMS = relapsing-remitting MS

Comparing the mild and moderate disability groups, SPMS was the strongest predictor of higher disability (HR 29.99; 95% CI 13.96–64.41; p<0.000). The total number of relapses was highly significant, but had a low impact on disability (HR 1.13; 95% CI 1.07–1.19; p<0.000). Complete remission of onset symptoms proved to be prognostic of a better long term outcome, thus reducing the risk of reaching even moderate disability to an HR of 0.42 (95% CI 0.23–0.77; p = 0.005).

The comparison of the mild and severe EDSS groups confirmed the strong correlation between SPMS and disability level (HR 503.8; 95% CI 160.0–1580.1; p<0.000). Onset pyramidal symptoms, development of depression and cognitive dysfunction were associated with a three- to four-fold risk of severe disability. OCB positivity and pregnancy did not show any association with disability outcome, neither in the overall regression model nor in the subgroup models.

## Discussion

In this observational study of a large and well-documented real-life cohort of RRMS patients we investigated the prognostic impact of clinical parameters on long term outcome as measured by the level of disability 10 years after disease onset.

### Occurrence of secondary progressive disease course is the most important negative predictor of long term outcome

We found that the occurrence of secondary progressive disease course is by far the most important negative predictor of long term outcome. A patient developing SPMS has a 500-fold higher risk of being severely disabled within 10 years after MS onset as compared to a patient remaining relapsing-remitting. This is consistent with findings of three large natural history studies (Lyon, Gothenborg and London, Ontario), although the overall rate of patients developing SPMS in our cohort was considerably lower [3,4,15]. This may be explained by the follow up period of 10 years from onset, which is likely to cause an underestimation of SPMS conversion rates.

### No association between OCB, higher age at onset, male sex and level of disability

A higher age at onset and male sex were associated with a shorter time to reach milestones of disability in those studies [3,4,15]. However, we could not find a significant correlation between sex and disability, similar to a more recently published study [5]. The different methodology and end points used may be responsible for this discrepancy, considering that other studies used survival analyses, while we used disability status after 10 years as an endpoint. We did find a significantly better outcome in patients with a younger age at onset, although–in contrast to other studies but consistent with recently published data from the Barcelona cohort–this result did not remain significant in the multinomial regression model [3,4,15,16]. Consequently, our results confirm that–although relevant–sex and age at onset only have a low impact on long term disability [16].

The Barcelona study also showed OCB positivity to be associated with disability accumulation in clinically isolated syndrome (CIS) [16]. However, the prognostic role of OCB in RRMS is far less clear [17]. We did not find any association between OCB and level of disability 10 years after disease onset suggesting that the prognostic value of OCB positivity regards more the prediction of conversion from CIS to clinically definite MS rather than in the prediction of a future disability accumulation.

It has been reported that relapses in the early MS course influence the long term outcome [4,18]. We could find a significant correlation between the total number of relapses and disability, although relapses accounted only for a minor increase to the overall risk of future disability. Consistent with prior studies, early relapses (from onset until year 5) contributed stronger to long term disability than late relapses (year 5 –year 10) [19,20]. While our results have to be interpreted cautiously in a cohort receiving heterogeneous DMT for variable time periods, they certainly confirm that relapses only play a minor part in the overall and long term accumulation of disability [21,22].

### Complete remission of onset symptoms predicts a favourable long term outcome

Complete remission of onset symptoms was identified to be predictive of a favourable long term outcome in both, the univariate and multivariate analysis. While only Kremenchutzky et al. could not find any effect of the degree of recovery on long term outcome, most natural history studies consistently showed incomplete recovery to be associated with a worse outcome regarding long term disability [4,22,23]. Irrespective of the immunopathological reasons for this favourable clinical outcome predictor–be it less severe inflammation along with less axonal dysfunction or a better capacity of repair/remyelination–our results confirm that the remission of onset symptoms provides a useful prognostic marker for RRMS patients in daily clinical practice.

### Depression and cognitive dysfunction are associated with disability–but pregnancy is not

In contrast to the general population, depression in MS is largely chronic suggesting a different pathophysiology [24]. Imaging studies support the view that depression in MS is due to structural brain abnormalities, most likely axonal damage resulting in grey matter atrophy [25–27]. Koch et. al. found depression unrelated to the development of SPMS, but this study was limited by a drop-out rate of 34% and the fact that it did not compare patients from disease onset [28].

Studies on cognitive dysfunction and disability have yielded controversial results. While some authors did not find any correlation between cognitive dysfunction and EDSS levels, others did [29–31]. Zivadinov et al. reported evidence hinting towards a link between cognitive deterioration and brain atrophy [32]. Of note, some studies found depression to be associated with cognitive dysfunction in patients with MS, whereas other investigations have not shown such a relationship [33–35].

To our knowledge, we provide evidence for the first time that clinically apparent depression and cognitive dysfunction are independent markers associated with a higher risk of accumulating disability in RRMS.

Previous studies on pregnancy and MS have demonstrated a reduction of relapse rates during pregnancy and an increase post-partum with a return to pre-pregnancy relapse rates within a short time [36,37]. However, information on pregnancies and their impact on the long term disease course and outcome are scant. In our study, we could not find any effect of pregnancy on future disability, neither negative nor positive, which is compatible with the above mentioned dissociation between relapses and irreversible disability.

The strengths of our study are the large number of patients and the close-meshed, standardized prospective follow up over a long term period of 10 years. As a limitation, it has to be acknowledged that the diagnosis of depression and cognitive dysfunction was primarily clinically based on repetitive exploration of patients for characteristic respective symptoms rather than performing standardized testing in every even asymptomatic patient. However, if cognitive dysfunction was suspected from exploration, formal neuropsychological testing was performed. In addition, we validated our clinical definitions of depression and cognitive dysfunction and found strong accordance rates compared to formal neuropsychological testing. Nevertheless, we cannot exclude that this thorough clinical routine practice may have led to underestimating the prevalence of depression and cognitive dysfunction. Since neuropsychological testing methods for depression and cognitive dysfunction changed significantly over the period of observation, we were not able to compare specific neuropsychological patterns of dysfunction. We also did not include fatigue in our analyses which may interfere with the clinical definitions of depression and cognitive dysfunction. Possible limitations also include interrater-variabilities of EDSS scoring, which is the primary endpoint of the study [38]. Still, due to extensive clinical trial engagements our physicians are well trained and certified as EDSS raters, thus minimizing this potential confounding factor. The EDSS is known to be rather crude in patients with low disability and is very much driven by walking disability, while factors such as cognition, fatigue, and depression, which play an important role in patients’ quality of life, are disregarded [39,40]. Due to the different group sizes (583 mildly, 132 moderately, and 78 severely disabled patients), the comparison of the different disability groups in the statistical analyses have to be treated with caution.

Considering the MS prevalence in the geographic area, our study seems to have caught most of its MS patients. Still, a potential selection bias towards more benign courses and/or severely disabled patients, who both tend to stop attending MS clinics, cannot be completely excluded.

Because of the low proportion of patients reaching high EDSS scores, we decided not to use time to EDSS scores (survival analysis) as an endpoint to avoid censor bias. However, by using disability categories we cannot describe the velocity of disease progression in our cohort. In addition, we focused on the strength of the detailed and confirmed clinical data, and did not include MRI data, because MRI was obtained at different institutions and not systematically performed. Finally, this study was not designed to show any DMT effects on the long term disease course. However, evidence confirming a true disease modifying treatment effect–namely decrease of conversion rates to SPMS—is scarce. This also underlines the importance of focusing future research on identifying biomarkers capable of characterizing the course of MS and developing therapeutic agents capable of reducing axonal degeneration in MS.

### Relevance for clinical routine practice

Apart from suggested predictive value of MRI parameters, the strongest clinical prognostic factors for substantial disability within 10 years after onset of MS regard the extent of early disease activity in terms of polysymptomatic onset symptoms, incomplete remission, relapse rate and early conversion to SPMS, as well as depression and development of cognitive dysfunction. These clinical prognostic factors thus can be easily determined and monitored in daily clinical routine practice and might be factored in for treatment decision-making.

## Supporting Information

S1 Table Correlation of clinical diagnosis of depression and cognitive dysfunction with formal neuropsychological testing. (DOCX)

S2 Table Disease modifying therapies and EDSS outcome after 10 years in ROMS patients. (DOCX)

## Abbreviations

ANOVA: Analysis of variance
ARR: Annualized relapse rates
CI: Confidence interval
CNS: Central nervous system
CSF: Cerebrospinal fluid
DMT: disease modifying therapy
EDSS: Expanded disability status scale
FS: Functional systems
HR: Hazard ratio
MS: Multiple sclerosis
MRI: Magnetic resonance imaging
OCB: Oligoclonal bands
PPMS: Primary progressive multiple sclerosis
RRMS: Relapsing remitting multiple sclerosis
SD: Standard deviation
SPMS: Secondary progressive multiple sclerosis
