# Supplementary material for: Long Term Clinical Prognostic Factors in Relapsing-Remitting Multiple Sclerosis: Insights from a 10-Year Observational Study
Source: PLoS One. 2016 Jul 8;11(7):e0158978. doi: 10.1371/journal.pone.0158978 (PMC4938610; doi:10.1371/journal.pone.0158978)
Supplement: S1 Table — (DOCX) [file pone.0158978.s001.docx]

**S1 Table. Correlation of clinical diagnosis of depression and cognitive dysfunction with formal neuropsychological testing.**

| **Depression** |  |  | **Formal testing for depression (n=140)** | |  |
| --- | --- | --- | --- | --- | --- |
|  |  |  | **No depression** | **depression** |  |
| **Clinical diagnosis of depression** | **No depression** | count | 48 | 5 |  |
|  |  | expected | 18.2 | 34.8 |  |
|  | **Depression** | count | 0 | 87 |  |
|  |  | expected | 29.8 | 57.2 |  |
| kappa |  |  |  |  | **0.923** |
| p-value |  |  |  |  | **<0.001** |
| **Cognitive dysfunction** |  |  | **Formal neuropsychological testing (n=86)** | |  |
|  |  |  | **No cognitive dysfunction** | **Cognitive dysfunction** |  |
| **Clinical diagnosis of cognitive dysfunction** | **No cognitive dysfunction** | count | 39 | 3 |  |
|  |  | expected | 20.0 | 22.0 |  |
|  | **Cognitive dysfunction** | count | 2 | 42 |  |
|  |  | expected | 21.0 | 23.0 |  |
| kappa |  |  |  |  | **0.884** |
| p-value |  |  |  |  | **<0.001** |
