# Supplementary material for: Long Term Clinical Prognostic Factors in Relapsing-Remitting Multiple Sclerosis: Insights from a 10-Year Observational Study
Source: PLoS One. 2016 Jul 8;11(7):e0158978. doi: 10.1371/journal.pone.0158978 (PMC4938610; doi:10.1371/journal.pone.0158978)
Supplement: S2 Table — (DOCX) [file pone.0158978.s002.docx]

**S2 Table. Disease modifying therapies and EDSS outcome after 10 years in ROMS patients.**

|  | **substance** | **Overall (n=793)** | **Mild disability (EDSS 0-2.5) (n=583)** | **Moderate disability (EDSS 3-5.5) (n=132)** | **Severe disability (EDSS 6-10) (n=78)** |
| --- | --- | --- | --- | --- | --- |
| **First DMT applied(n=593)** | Interferonß1a i.m | 168 (28.3%) | 133 (32.8%) | 25 (20.7%) | 10 (15.2%) |
|  | Interferonß1a s.c | 110 (18.5%) | 80 (19.7%) | 19 (15.7%) | 11 (16.7%) |
|  | Interferonß1b s.c. | 138 (23.3%) | 96 (23.6%) | 27 (22.3%) | 15 (22.7%) |
|  | Glatirameracetat | 97 (16.4%) | 75 (18.5%) | 15 (12.4%) | 7 (10.6%) |
|  | Fingolimod | 17 (2.9%) | 9 (2.2%) | 6 (5.0%) | 2 (3.0%) |
|  | Natalizumab | 5 (0.8%) | 4 (1.0%) | 1 (0.8%) | 0 (0.0%) |
|  | Azathioprine | 55 (9.3%) | 6 (1.5%) | 28 (23.1%) | 21 (31,8%) |
|  | other | 3 (0.5%) | 3 (0.7%) | 0 (0.0%) | 0 (0.0%) |
| **Second DMT applied (n=365)** | Interferonß1a i.m | 54 (14.8%) | 40 (17.5%) | 11 (17.5%) | 3 (6.1%) |
|  | Interferonß1a s.c. | 47 (12.9%) | 28 (12.2%) | 12 (12.2%) | 7 (14.3%) |
|  | Interferonß1b s.c. | 39 (10.7%) | 21 (9.2%) | 13 (9.2%) | 5 (10.2%) |
|  | Glatirameracetat | 54 (14.8%) | 40 (17.5%) | 9 (17.5%) | 5 (10.2%) |
|  | Fingolimod | 31 (8.5%) | 20 (8.7%) | 4 (8.7%) | 7 (14.3%) |
|  | Natalizumab | 80 (21.9%) | 54 (23.6%) | 21 (23.6%) | 5 (10.2%) |
|  | Cyclophosphamide | 15 (4.1%) | 8 (3.5%) | 4 (3.5%) | 3 (6.1%) |
|  | Mitoxantrone | 12 (3.3%) | 2 (0.9%) | 4 (0.9%) | 6 (12.2%) |
|  | Azathioprine | 20 (5.5%) | 9 (3.9%) | 6 (3.9%) | 5 (10.2%) |
|  | other | 13 (3.6%) | 7 (3.1%) | 3 (3.1%) | 3 (6.1%) |
| **Third DMT applied (n=201)** | Interferonß1a i.m | 19 (9.5%) | 11 (9.9%) | 6 (10.2%) | 2 (6.5%) |
|  | Interferonß1a s.c. | 19 (9.5%) | 13 (11.7%) | 5 (8.5%) | 1 (3.2%) |
|  | Interferonß1b s.c. | 19 (9.5%) | 13 (11.7%) | 3 (5.1%) | 3 (9.7%) |
|  | Glatirameracetat | 19 (9.5%) | 12 (10.8%) | 4 (6.8%) | 3 (9.7%) |
|  | Fingolimod | 6 (3.0%) | 6 (5.4%) | 0 (0.0%) | 0 (0.0%) |
|  | Natalizumab | 19 (9.5%) | 11 (9.9%) | 8 (13.6%) | 0 (0.0%) |
|  | IVIG | 49 (24.4%) | 25 (22.5%) | 15 (25.4%) | 9 (29.0%) |
|  | Cyclophosphamide | 16 (8.0%) | 2 (1.8%) | 10 (16.9%) | 4 (12.9%) |
|  | Mitoxantrone | 7 (3.5%) | 2 (1.8%) | 1 (1.7%) | 4 (12.9%) |
|  | Azathioprine | 17 (8.5%) | 10 (9.0%) | 5 (8.5%) | 2 (6.5%) |
|  | other | 11 (5.5%) | 6 (5.4%) | 2 (3.4%) | 3 (9.7%) |
| **Fourth DMT applied (n=111)** | Interferonß1a i.m | 5 (4.5%) | 5 (8.3%) | 2 (5.9%) | 0 (0.0%) |
|  | Interferonß1a s.c. | 9 (8.1%) | 3 (5.0%) | 0 (0.0%) | 4 (23.5%) |
|  | Interferonß1b | 3 (2.7%) | 2 (3.3%) | 1 (2.9%) | 0 (0.0%) |
|  | Glatirameracetat | 10 (9.0%) | 8 (13.3%) | 2 (5.9%) | 0 (0.0%) |
|  | Fingolimod | 6 (5.4%) | 2 (3.3%) | 3 (8.8%) | 1 (5.9%) |
|  | Natalizumab | 20 (18.0%) | 13 (21.7%) | 7 (20.6%) | 0 (0.0%) |
|  | IVIG | 24 (21.6%) | 13 (21.7%) | 6 (17.6%) | 5 (29.4%) |
|  | Cyclophosphamide | 10 (9.0%) | 3 (5.0%) | 5 (14.7%) | 2 (11.8%) |
|  | Mitoxantrone | 5 (4.5%) | 0 (0.0%) | 4 (11.8%) | 1 (5.9%) |
|  | Azathioprine | 11 (9.9%) | 7 (11.7%) | 1 (2.9%) | 3 (17.6%) |
|  | other | 8 (7.2%) | 4 (6.7%) | 3 (8.8%) | 1 (5.9%) |
| **Fifth DMT applied (n=46)** | Interferonß1a i.m | 4 (8.7%) | 3 (13.6%) | 1 (6.7%) | 0 (0.0%) |
|  | Interferonß1a s.c. | 2 (4.3%) | 2 (9.1%) | 0 (0.0%) | 0 (0.0%) |
|  | Interferonß1b | 4 (8.7%) | 1 (4.5%) | 2 (13.3%) | 1 (11.1%) |
|  | Glatirameracetat | 4 (8.7%) | 2 (9.1%) | 2 (13.3%) | 0 (0.0%) |
|  | Fingolimod | 6 (13.0%) | 4 (18.2%) | 2 (13.3%) | 0 (0.0%) |
|  | Natalizumab | 4 (8.7%) | 3 (13.6%) | 1 (6.7%) | 0 (0.0%) |
|  | IVIG | 7 (15.2%) | 3 (13.6%) | 1 (6.7%) | 3 (33.3%) |
|  | Cyclophosphamide | 6 (13.0%) | 2 (9.1%) | 3 (20.0%) | 1 (11.1%) |
|  | Mitoxantrone | 3 (6.5%) | 2 (9.1%) | 1 (6.7%) | 0 (0.0%) |
|  | Azathioprine | 2 (4.3%) | 0 (0.0%) | 0 (0.0%) | 2 (22.2%) |
|  | other | 4 (8.7%) | 0 (0.0%) | 2 (13.3%) | 2 (22.2%) |
| **Sixth DMT applied (n=22)** | Interferonß1a i.m | 1 (4.5%) | 0 (0.0%) | 1 (12.5%) | 0 (0.0%) |
|  | Interferonß1a s.c. | 1 (4.5%) | 1 (11.1%) | 0 (0.0%) | 0 (0.0%) |
|  | Fingolimod | 2 (9.1%) | 1 (11.1%) | 1 (12.5%) | 0 (0.0%) |
|  | Natalizumab | 3 (13.6%) | 3 (33.3%) | 0 (0.0%) | 0 (0.0%) |
|  | IVIG | 7 (31.8%) | 1 (11.1%) | 4 (50.0%) | 2 (40.0%) |
|  | Cyclophosphamide | 2 (9.1%) | 1 (11.1%) | 1 (12.5%) | 0 (0.0%) |
|  | Azathioprine | 3 (13.6%) | 2 (22.2%) | 0 (0.0%) | 1 (20.0%) |
|  | other | 3 (13.6%) | 0 (0.0%) | 1 (12.5%) | 2 (40.0%) |
